# Recurrent *LRP1-SNRNP25* and *KCNMB4-CCND3* fusion genes promote tumor cell motility in human osteosarcoma

**DOI:** 10.1186/s13045-014-0076-2

**Published:** 2014-10-10

**Authors:** Jilong Yang, Matti Annala, Ping Ji, Guowen Wang, Hong Zheng, David Codgell, Xiaoling Du, Zhiwei Fang, Baocun Sun, Matti Nykter, Kexin Chen, Wei Zhang

**Affiliations:** Department of Bone and Soft Tissue Tumor, Tianjin Medical University Cancer Hospital & Institute, Tianjin, 30060 PR China; Department of Signal Processing, Tampere University of Technology, Tampere, 33101 Finland; Institute of Biomedical Technology, University of Tampere, Tampere, 33520 Finland; Department of Pathology, University of Texas MD Anderson Cancer Center, Houston, TX 77030 USA; Department of Epidemiology and Biostatistics, Tianjin Medical University Cancer Hospital & Institute, Tianjin, 30060 PR China; Department of Pathology, Tianjin Medical University Cancer Hospital & Institute, Tianjin, 30060 PR China; Department of Bone and Soft Tissue Tumors, Beijing University Cancer Hospital, Beijing, 100020 PR China; National Clinical Research Center of Cancer, Tianjin Medical University Cancer Institute and Hospital, Tianjin, 300060 PR China; Department of Diagnostics, Tianjin Medical University, Tianjin, 30060 PR China

**Keywords:** Osteosarcoma, Transcriptome sequencing, Fusion gene, *LRP1-SNRNP25*, *KCNMB4-CCND3*

## Abstract

**Background:**

The identification of fusion genes such as *SYT-SSX1/SSX2, PAX3-FOXO1, TPM3/TPM4-ALK* and *EWS-FLI1* in human sarcomas has provided important insight into the diagnosis and targeted therapy of sarcomas. No recurrent fusion has been reported in human osteosarcoma.

**Methods:**

Transcriptome sequencing was used to characterize the gene fusions and mutations in 11 human osteosarcomas.

**Results:**

Nine of 11 samples were found to harbor genetic inactivating alterations in the *TP53* pathway. Two recurrent fusion genes associated with the 12q locus, *LRP1-SNRNP25* and *KCNMB4-CCND3,* were identified and validated by RT-PCR, Sanger sequencing and fluorescence *in situ* hybridization, and were found to be osteosarcoma specific in a validation cohort of 240 other sarcomas. Expression of *LRP1-SNRNP25* fusion gene promoted SAOS-2 osteosarcoma cell migration and invasion. Expression of *KCNMB4-CCND3* fusion gene promoted SAOS-2 cell migration.

**Conclusions:**

Our study represents the first whole transcriptome analysis of untreated human osteosarcoma. Our discovery of two osteosarcoma specific fusion genes associated with osteosarcoma cellular motility highlights the heterogeneity of osteosarcoma and provides opportunities for new treatment modalities.

**Electronic supplementary material:**

The online version of this article (doi:10.1186/s13045-014-0076-2) contains supplementary material, which is available to authorized users.

## Background

Among solid tumors, sarcomas were the first cancer type associated with chromosomal translocations and gene fusions [1]. Approximately 15–20% of sarcomas in about 15 sarcoma types, including Ewing sarcoma, synovial sarcoma, desmoplastic small round cell tumor, alveolar rhabdomyosarcoma, dermatofibrosarcoma protuberans and myxoid liposarcoma have been found to harbor specific gene fusions [2-5]. The identification of fusion genes such as *SYT-SSX1/SSX2, PAX3-FOXO1, TPM3/TPM4-ALK, BCOR-CCNB3* and *EWS-FLI1* in human sarcomas has provided important insight into the diagnosis and targeted therapy of sarcomas [6-10]. However, no recurrent fusion has ever been found in human osteosarcoma, although osteosarcoma is known to exhibit frequent numerical and structural chromosomal aberrations, such as *TP53* mutations and deletions, *MDM2* amplification, *CDKN2A* deletion, and hemi- or homozygous loss of *RB1* [5,11,12].

A major challenge in the molecular study of osteosarcoma is the difficulty of obtaining sufficient quantities of fresh untreated tumor tissue, since neoadjuvant chemotherapy is often used prior to surgery in osteosarcoma. Here we report the first transcriptome sequencing study of untreated osteosarcoma. Two selectively rearranged genomic loci that gave rise to fusion genes in 5 of 11 tumors were detected. One hotspot in 17p associated with *TP53*-disrupting rearrangements, while the other hotspot in 12q associated with 12q amplification. Two recurrent fusion genes associated with the 12q locus, *LRP1-SNRNP25* and *KCNMB4-CCND3,* were validated and investigated. Our discovery of novel osteosarcoma fusion genes and rearrangement hotspots provides important insight into the role that chromosomal rearrangements play in p53 pathway inactivation and regulation of cell motility in osteosarcoma cells.

## Results

### Transcriptome sequencing cohort

Sarcoma tissue and information collection for this study at Tianjin Medical University Cancer Institute & Hospital (TMUCIH) was performed according to the protocol approved by the Institutional Review Board (IRB) of TMUCHIH and with patient consent. We acquired primary tumor tissue from 31 untreated osteosarcoma biopsies obtained from the Tumor Tissue Bank (TTB) at TMUCIH. A sufficient quantity of high quality RNA was obtained from 11 of 31 cases (10 conventional subtype and 1 parosteal subtype). The cohort included 6 male and 5 female patients between 16 and 48 years of age (Additional file 1: Table S1). Extracted whole RNA was sequenced using IlluminaHiSeq™ 2000 instruments at BGI. Sequence quality was high in all samples, with 30% of coding regions covered by 10× or higher coverage (Additional file 1: Figure S1). All Spearman correlations between sample gene expression profiles were above 0.85.

### Identification of two fusion gene hotspots using transcriptome sequencing

**Figure 1:**
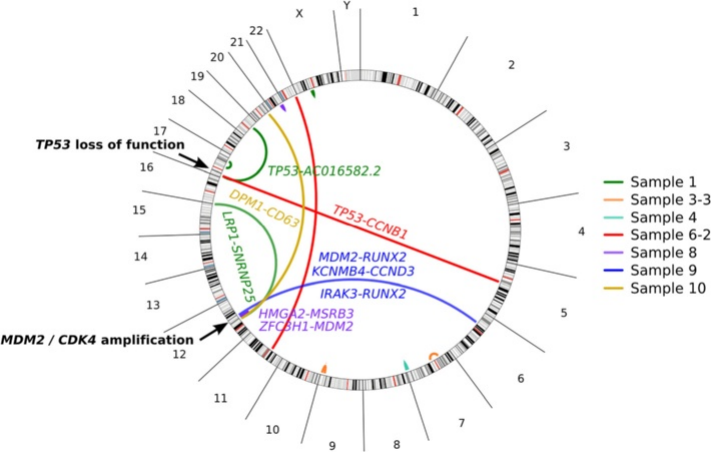
**Transcriptome sequencing of 11 osteosarcomas revealed two hotspots of chromosomal rearrangement.** One hotspot in 17p was associated with *TP53*-disrupting rearrangements. The second hotspot in 12q was associated with *MDM2/CDK4* co-amplification.

Fusion gene detection based on transcriptome sequencing identified a total of 16 fusion genes in our cohort of 11 osteosarcomas. 7 of 11 osteosarcomas harbored at least one fusion gene (Additional file 1: Figure S2, Tables S1 and S2). We identified a pattern of interchromosomal gene fusions clustered at two hotspots in the genome (Figure 1). The first hotspot, rearranged in two osteosarcomas (samples 1 and 6-2), coincided with the *TP53* tumor suppressor gene in 17p. Sample 6-2 harbored a fusion between *TP53* and *cyclinB1(CCNB1)*, while sample 1 had *TP53* juxtaposed with the non-coding gene *AC016582.2*, whose biological function is unknown. Both fusions disrupted *TP53* between exons 1 and 2, preventing the production of p53 protein from one allele (Additional file 1: Figure S3). Based on the fusion transcript structure, neither *TP53* rearrangement is expected to produce chimeric protein.

**Figure 2:**
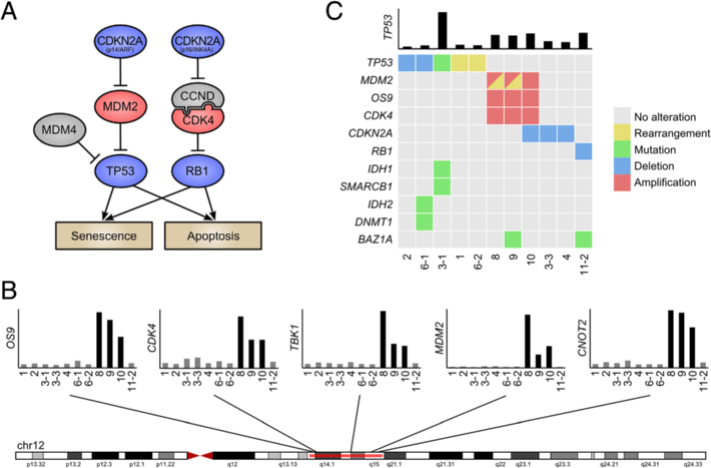
**Recurrent TP53 pathway alterations in human osteosarcoma. (A)** Activating (red) and inactivating (blue) alterations in the p53 and RB1 pathways identified in our cohort. **(B)** Samples 8, 9, 10 harbored 12q co-amplification of *CDK4* and *MDM2*. 12q amplification was mutually exclusive with alterations in the *TP53* gene. **(C)** Matrix of genetic alterations observed in our cohort. Nine of 11 patients harbored disrupting alterations in the *TP53* pathway genes.

The second hotspot, located in 12q, was rearranged in four osteosarcomas (samples 1, 8, 9, 10) and coincided with the genes *MDM2* and *CDK4* (Figure 1). *MDM2* and *CDK4* are known to be frequently co-amplified in osteosarcoma and contribute to suppression of the p53 and RB1 pathways (Figure 2A) [12-14]. A strong localized gene dosage effect was observed in three of the four 12q-rearranged cases (samples 8, 9 and 10), suggesting that fusion genes in this locus often arise as a by-product of *MDM2/CDK4* co-amplification (Figure 2B). Many of the 12q fusion genes produced chimeric proteins or disrupted cancer-associated genes such as *RUNX2*, *CCND3*, and *LRP1*, indicating that some of the fusions may contribute to cancer progression independently of *MDM2/CDK4* co-amplification.

### Widespread alteration of p53 and RB1 pathways in osteosarcoma

Based on the observed rearrangements in the *TP53* and *MDM2/CDK4* loci, we set out to analyze the p53 and RB1 pathways for further alterations. We used the transcriptome sequencing data to look for oncogenic mutations in expressed regions of the genome, and performed a gene dosage analysis to identify signs of copy number alterations. In addition to the two *TP53*-rearranged samples (sample 1 and 6-2), we discovered a *TP53* mutation with loss-of-heterozygosity in one sample (sample 3-1) (Figure 2C, Additional file 1: Figure S4), and *TP53* deletions in two samples (samples 2 and 6-1) (Figure 2C, Additional file 1: Figure S5). Of the six remaining osteosarcomas with intact *TP53*, three carried *MDM2/CDK4* co-amplification (samples 8, 9, and 10). Three samples had lost *CDKN2A* (samples 3-3, 4, and 10), and one showed signs of *RB1* loss (sample 11-2) (Figure 2C). Taken together, all 11 osteosarcomas in our cohort had lost either p53 or RB1 pathway function through one of these mechanisms (Figure 2C).

Our mutation analysis revealed 522 non-synonymous variants, including mutations in genes *IDH1*, *IDH2*, *SMARCB1*, *DNMT1*, *BRD7* and *PIK3C3* (Figure 2C). The *IDH1* R132H mutation found in osteosarcoma sample 3-1 is a frequent event in low-grade brain tumors, central and periosteal chondromas, and central chondrosarcomas [15,16]. Sample 3-1 also harbored a non-synonymous mutation in *SMARCB1*, a gene that has been associated with congenital risk of rhabdoid tumors and chondrosarcomas [17]. Histopathological analysis of neoplastic cells from sample 3-1 revealed that they resided in lacunar spaces surrounded by hyaline matrix and displayed a chondrocytic phenotype with severe cytological atypia, confirming the diagnosis of chondroblastic osteosarcoma.

### Recurrent *LRP1-SNRNP25* and KCNMB4-CCND3 fusion genes in osteosarcoma

**Figure 3:**
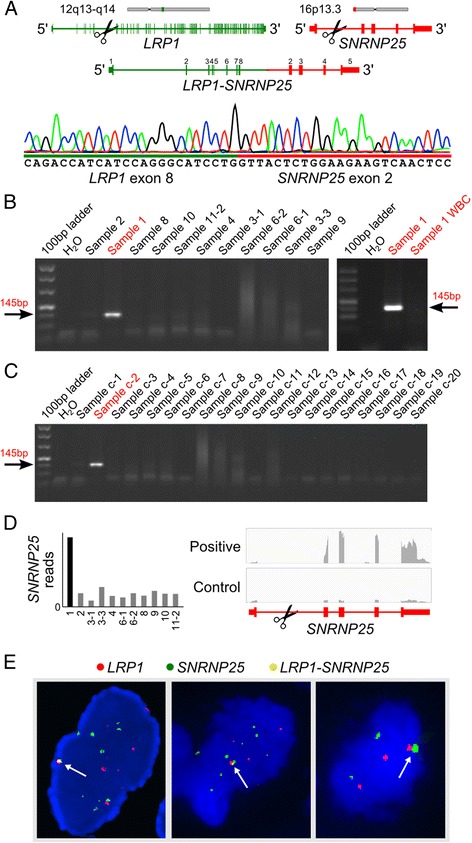
**Validation and identification of fusion genes. (A)** Structure of the *LRP1-SNRNP25* fusion gene. Sanger sequencing of the fusion junction is shown at the bottom. **(B)** RT-PCR validation of fusion transcript in the sequenced cohort. Matched normal white blood cells (WBC) were negative for fusion. **(C)** RT-PCR identified a second fusion positive case in a validation cohort of 20 osteosarcomas. **(D)** Fusion leads to elevated expression of *SNRNP25* exons 2-5. **(E)** Interchromosomal rearrangement juxtaposing *LRP1* and *SNRNP25* was validated using fluorescence in situ hybridization (FISH). Arrows indicate overlapping probes.

We selected the fusion genes *LRP1-SNRNP25, KCNMB4-CCND3, MDM2-RUNX2, TP53-CCNB1, DPM1-CD63* and *ZFC3H1-MDM2* for further validation as they involved genes known to be involved in cancer progression. All six fusion genes were successfully validated in the sequencing cohort using RT-PCR and Sanger sequencing targeted at fusion junctions (Figure 3A-C, Additional file 1: Table S3, Figures S6–S11). We then used the same RT-PCR assay on 20 additional osteosarcoma biopsies that did not yield enough RNA for whole transcriptome sequencing (17 conventional, 2 parosteal, and 1 low grade central subtype). Among the total 31 cases, only the *LRP1-SNRNP25* and *KCNMB4-CCND3* fusion genes were found to be recurrent, each found in 2 of 31 cases (6.5%) (Figure 3B-C, Additional file 1: Figure S6). Matched normal white blood cells were negative for fusion transcripts, demonstrating the somatic origin of the fusion genes (Figure 3B, Additional file 1: Figure S6–S11).

To determine whether the *LRP1-SNRNP25* and *KCNMB4-CCND3* fusions were specific to osteosarcomas, we repeated our RT-PCR analysis on 240 fresh tissues from 8 other sarcoma subtypes, including 56 malignant fibrous histiocytoma/undifferentiated pleomorphic sarcoma (MFH/UPS), 50 liposarcomas, 24 soft tissue leiomyosarcomas, 4 rhabdomyosarcomas, 21 synovial sarcomas, 13 chondrosarcoma, 8 EWS/PNETs, and 64 malignant peripheral nerve sheath tumors (MPNSTs) (Additional file 1: Table S4). None of the 240 other sarcomas was positive for *LRP1-SNRNP25* or *KCNMB4-CCND3* fusions, nor for the four other fusion genes found in our analysis.

In both *LRP1-SNRNP25* positive tumors, the fusion juxtaposed exon 8 of *LRP1* with exon 2 of *SNRNP25* (Figure 3A) and resulted in increased expression of *SNRNP25* under control of the *LRP1* promoter (Figure 3D). The fusion was in-frame and produced a chimeric protein that merged the first 409 amino acids of LRP1’s extracellular domain with the ubiquitin-like domain (amino acids 24-132) of SNRNP25.

Since *LRP1* and *SNRNP25* are normally located on different chromosomes, we set out to validate the interchromosomal rearrangement using FISH. We used bacterial artificial chromosome (BAC) probes to label the two genes in frozen sections from the two positive cases. More than 90% cells displayed *LRP1* probes, *SNRNP25* probes and the overlapping probes, validating the rearrangement (Figure 3E). The FISH also revealed amplification of both *LRP1* and *SNRNP25*, suggesting a complex genetic alteration of osteosarcoma. Similar FISH assays validated the *KCNMB4-CCND3* rearrangement (Additional file 1: Figure S6D).

In the *KCNMB4-CCND3* positive cases, the fusion juxtaposed exon 1 of *KCNMB4* with exon 4 of *CCND3* (Additional file 1: Figure S6). The fusion was in-frame and produced a chimeric protein that merged the cytoplasmic and transmembrane domains (112 amino acids) of KCNMB4 with amino acids 67-292 of CCND3. Most sarcoma-associated fusion genes such as *SYT-SSX1/SSX2, BCOR*-*CCNB3, EWS-FLI1*, *PAX3-FOXO1* and *COL1A1-PDGFB* produce functional proteins [6-9,18]. We sought to detect chimeric *KCNMB4-CCND3* and *LRP1-SNRNP25* protein products in 31 human osteosarcoma tissues by western blotting with antibodies against CCND3, KCNMB4, SNRNP25 and LRP1. Unfortunately our antibodies detected no evidence of fusion proteins in either the RT-PCR positive or negative cases. To investigate chimeric protein products in tissue sections, we analyzed protein expression of LRP1, SNRNP25, KCNMB4 and CCND3 in 4 fusion positive and 27 fusion negative osteosarcomas with the same antibodies, but found no significant difference in protein expression between fusion positive and negative cases.

### *LRP1-SNRNP25* and *KCNMB4-CCND3* contribute to tumor cell motility

To study any oncogenic contribution conferred by fusion genes in our cohort, we cloned the *LRP1-SNRNP25* and *KCNMB4-CCND3* fusion genes into pcDNA3.1 expression vectors and validated the vectors by sequencing (Additional file 1: Figure S12A). Transfection of the *LRP1-SNRNP25* and *KCNMB4-CCND3* fusion genes into human osteosarcoma SAOS-2 cells resulted in detectable expression of fusion proteins (Additional file 1: Figure S12B).

**Figure 4:**
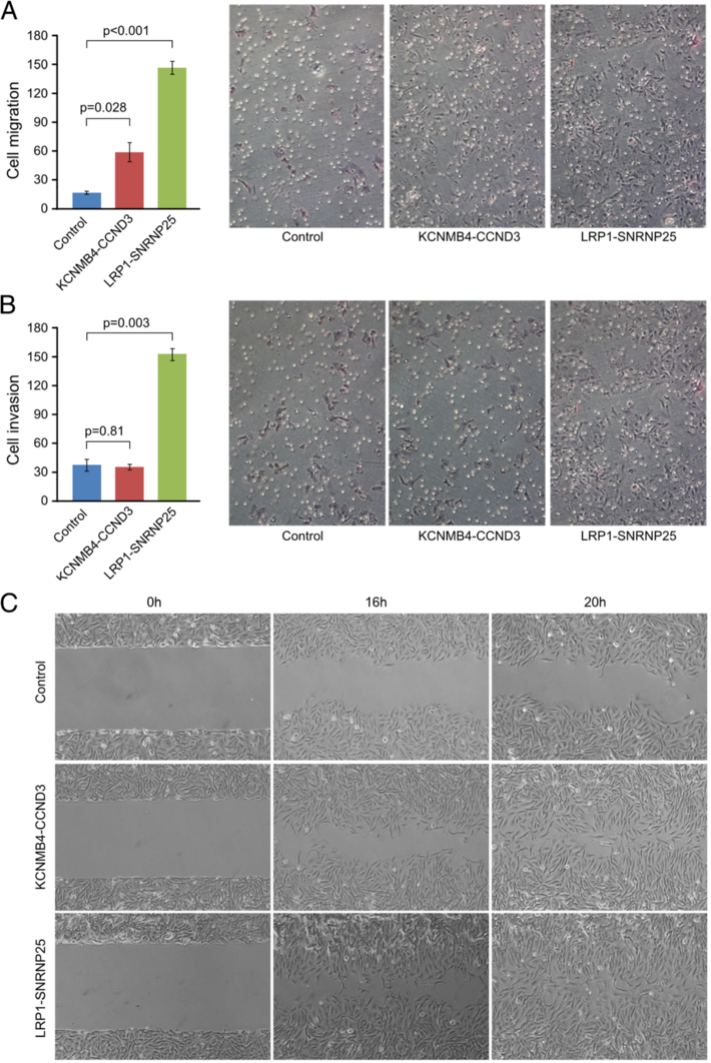
***LRP1-SNRNP25*** **and** ***KCNMB4-CCND3*** **fusion genes promoted human osteosarcoma SAOS-2 cells motility relative to empty vector. (A)** Transwell migration assay. Both the *LRP1-SNRNP25* and *KCNMB4-CCND3* fusions significantly promoted cell migration of SAOS-2 cells. **(B)** Transwell invasion assay. While the *LRP1-SNRNP25* promoted invasion of SAOS-2 cells significantly, *KCNMB4-CCND3* fusions did not significantly promote cell invasion. **(C)** Wound healing assay showed that both fusions accelerated the osteosarcoma cell migration.

Since *LRP1* and *CCND3* play important roles in regulating tumor cell migration, invasion, proliferation and apoptosis in other cancers [19-22], we set out to test whether the chimeric proteins might play any role in osteosarcoma cells. A cell transformation assay with Rat2 fibroblast cells revealed that neither fusion was sufficient to transform Rat2 fibroblast cells (Additional file 1: Figure S12C) or to augment K-Ras V12 driven cell transformation (Additional file 1: Figure S12D). In soft agar colony formation assays, neither fusion promoted anchorage-independent colony formation relative to GFP controls (Additional file 1: Figure S12E). Proliferation of SAOS-2 cells was inhibited rather than increased after fusion transfection (Additional file 1: Figure S12F). However, both the *LRP1-SNRNP25* and *KCNMB4-CCND3* fusions significantly promoted cell migration of SAOS-2 cells in transwell assays (Figure 4A), and *LRP1-SNRNP25* also promoted invasion significantly (Figure 4B). A wound healing assay corroborated these findings, showing that both fusions accelerated cell migration (Figure 4C). Clinically, both *LRP1-SNRNP25* positive patients had tumor recurrence 21 months after surgery, and one of the patients developed lung metastases after 6 months. One of the *KCNMB4-CCND3* positive patients also had his tumor recur 6 months after surgery.

## Discussion

Osteosarcoma is the most common histological form of primary bone sarcoma and predominantly inflicts children and young adults. While it is often characterized as a genomically unstable bone sarcoma, no recurrent gene translocation and fusion genes has ever been reported in human osteosarcoma, except for one report which revealed exon 6 of the *cAMP-responsive element binding protein 3-like 1* gene (*CREB3L1*) fused in-frame to the *EWSR1* exon 11 in a case of small cell osteosarcoma [23].The most important discovery of the present study is the detection of two recurrent fusion genes, *LRP1-SNRNP25* and *KCNMB4-CCND3,* which are validated by RT-PCR, Sanger sequencing and FISH. Furthermore, preliminary functional studies show that expression of *LRP1-SNRNP25 and KCNMB4-CCND3* fusion genes promotes SAOS-2 osteosarcoma cell migration, while LRP1-SNRNP25 expression also promotes invasion. Taken together, these data suggest that *LRP1-SNRNP25* and *KCNMB4-CCND3* fusions confer an oncogenic effect in human osteosarcoma by enhancing cancer cell motility.

Further investigation into the functional roles of *KCNMB4-CCND3* and *LRP1-SNRNP25* is necessary, particularly to investigate the mechanisms through which the fusion genes affect invasion/migration. *LRP1* encodes a membrane-bound receptor that has been implicated in tumor cell invasion, migration, proliferation, and apoptosis [24,25]. LRP1 forms a signaling complex with PDGFR-beta in endosomes and regulates activation of the MAPK pathway [24]. Furthermore, LRP1 promotes anti-apoptotic signaling in neurons by activating the AKT survival pathway [25]. In healthy cells, cyclin D3 (CCND3) regulates the G1/S cell cycle transition by phosphorylating RB1 in complex with cyclin dependent kinases 4 and 6 (CDK4/CDK6). Regulated by HDAC5, miR-195, miR-138, and miR-16 family, CCND3 triggers an accumulation of cells in S phase [19-22]. In GIST and glioblastoma, CCND3 has been shown to be a key target of miR-195-induced inhibition of cell invasion [19-22]. Thus, the mechanisms through which these two *KCNMB4-CCND3* and *LRP1-SNRNP25* fusion genes may affect invasion/migration appear to be complex. Our preliminary data shows that the MMP-2, MMP-9, p-AKT, pERK, pMAPK, and JNK were increased significantly in transfected SAOS-2 tumor cells, while caspase-3 and Ki-67 did not change significantly (data not shown). These results suggest that the fusion events might promote tumor cell invasion and migration by elevating MMPs and activating the AKT and MAPK signaling pathways. Future studies will be needed to shed light on the driving mechanisms.

The recurrent fusion genes *LRP1-SNRNP25* and *KCNMB4-CCND3* found in this study both involved a 5′ partner gene located near the *MDM2/CDK4* locus in 12q. This suggests that the fusion genes arise as a by-product of the chromosomal rearrangements that result in *MDM2/CDK4* co-amplification. However, the recurrent nature of these fusions suggests that they are selected for during osteosarcoma development. This hypothesis is supported by the observation that all four *LRP1-SNRNP25* and *KCNMB4-CCND3* fusions had a transcript structure compatible with production of chimeric protein. Fusion genes that arise as a by-product of somatic copy number alterations but contribute an independent oncogenic effect have been observed in other cancers. In glioblastoma, *EGFR-SEPT14* and *EGFR-PSPH* fusions associated with *EGFR* amplification were recently observed to result in constitutive activation of EGFR signaling [26,27].

One limitation of the current study is the limited cohort size of 31 osteosarcomas. We hope to move forward by collaborating with other cancer centers and research groups that possess more untreated or treated osteosarcoma samples. Another limitation is that we did not identify the genomic breakpoints and protein products associated with *KCNMB4-CCND3* and *LRP1-SNRNP25* fusions in clinical samples. In the future we will perform whole genome sequencing of the fusion gene positive cases to define the exact genetic breakpoints. Another limitation is the fact that the fusion are low frequency events. This may reflect the high genetic heterogeneity of osteosarcomas.

## Conclusion

The present study represents the first whole transcriptome sequencing study of untreated human osteosarcoma. Our discovery of two osteosarcoma specific fusion genes associated with cell motility may provide opportunities for new treatment modalities. The findings of our study support the view that the majority of osteosarcomas harbor alterations in the p53 pathway, including recurrent translocations disrupting the *TP53* gene. Future studies with an expanded cohort will determine how frequent these events are and whether these gene fusions can potentially serve as therapeutic targets in future clinical practice.

## Material and methods

### Samples and RNA extraction and quality control

Sarcoma tissue and information collection for this study at Tianjin Medical University Cancer Institute & Hospital (TMUCIH) was performed according to the protocol approved by the Institutional Review Board (IRB) of TMUCHIH and with patient consent. We obtained 31 untreated osteosarcoma tissues from the Tumor Tissue Bank (TTB) at TMUCIH. All samples had at least 90% tumor content. Tumors were snap frozen in liquid nitrogen. After crushing tumors, we isolated total RNA using the TRIzol reagent kit (Invitrogen) that employs a method based on GITC-phenol-chloroform extraction. RNA was quantified with Qubit (Invitrogen) and Nanodrop ND1000 (ThermoFisher Scientific) before quality assessment with the Agilent 2100 Bioanalyzer. For blood white cell RNA isolation, we used the same method. Extracted whole RNA was sequenced using IlluminaHiSeq™ 2000 at the Beijing Genomics Institute (BGI).

### RNA library construction

PolyA mRNA was purified from 10 μg of total RNA using NucleoTrap mRNA (Macherey Nagel) according to the manufacturer’s protocol. Following the IlluminaHiSeq™ 2000 total RNA Seq kit instructions, we fragmented 100 ng of poly-A RNA by incubation with RNAse III for 10 min in a 10 μl reaction volume containing 1× RNAse III buffer with the enzyme. Fragmented RNA was then purified using the RiboMinus Concentration Module (Invitrogen). The yield and size distribution of the fragmented RNA was assessed using the Quant-iT RNA assay kit with the Qubitfluorometer (Invitrogen) and the RNA 6000 Pico Chip kit with the Agilent 2100 Bioanalyzer. A total of 50 ng of fragmented RNA was hybridized and ligated with the SOLiD adaptor mix and reverse transcribed according to the manufacturer’s instructions.

The isolated cDNA was size selected to be ~200 bp using Novex pre-cast gel products. The cDNA was then amplified according to the IlluminaHiSeq™ 2000 Total RNA Seq kit protocol. The yield and size distribution of the cDNA were assessed using the Quant-iT HS DNA assay kit with the Qubitfluorometer and the High Sensitivity DNA Assay Chip kit on the Agilent 2100 Bioanalyzer.

### Emulsion PCR and whole transcriptome paired-end sequencing

Beads with oligo(dT) were used to isolate poly(A) mRNA after total RNA was collected. Fragmentation buffer was used to cleave mRNA into short fragments. Taking these short fragments as templates, random hexamer primers were used to synthesize the first-strand cDNA. Second-strand cDNA was synthesized using buffer, dNTPs, RNase H and DNA polymerase I. Short fragments were purified with QiaQuick PCR extraction kit and resolved with EB buffer for end reparation and poly(A) addition. The short fragments were then ligated with sequencing adaptors. Agarose gel electrophoresis was used to select fragments suitable for amplification with PCR. The templated beads from each sample were deposited on two quadrants of a slide. Massively parallel ligation sequencing was carried out using IlluminaHiSeq™ 2000 instruments at the Beijing Genomics Institute following the manufacturer’s instructions. Each sequencing run produced approximately 50 million paired end reads where each mate was 90 bp in length.

### Gene and exon expression analysis

Whole transcriptome sequencing reads were aligned against the GRCh37 human reference genome using Tophat version 2.0.4 [28]. The number of overlapping reads was calculated for all exons and then for all genes annotated in Ensembl 67. Gene expression values were normalized across samples using median-of-ratios normalization. In this normalization method, expression profiles are normalized by calculating an expression ratio between two samples for every gene (or exon), and then taking the median of those ratios. All gene expression values are then multiplied by the median-of-ratios. Only genes covered by 500 or more total reads were used in calculating the median-of-ratios.

### Fusion gene detection

To achieve robust results, fusion gene discovery was performed using two different strategies [29]. In the first strategy, we applied the ChimeraScan fusion gene detection software by Iyer et al. to the raw FASTQ format sequencing data [30]. ChimeraScan used Bowtie 0.12.8 for read alignment [31]. Anchor length was set to 25 bp. One nucleotide mismatch was allowed in the initial alignments and in the alignment of discordant reads. Fusion gene candidates with less than 20 spanning reads were filtered out in order to focus the analysis on strongly expressed fusion genes.

The second strategy was to use an unpublished in-house fusion detection algorithm called Breakfast to validate the ChimeraScan results and to search for fusion genes where the fusion junction did not occur on an exon-exon junction, but instead disrupted an exon. The Breakfast algorithm operates on aligned SAM files, and therefore we first aligned whole transcriptome sequencing reads against the GRCh37 human reference genome using Tophat version 2.0.4 [28]. Breakfast searched the alignment data for discordant read pairs and unaligned individual mates. For discordant read pairs, we required the mates to be at least one megabase apart. The alignment quality of both mates in a discordant pair was required to be above 15 (phred). Next, individual unaligned mates were split into two 25 bp anchors that were extracted from both ends of each 90 bp mate. The 25 bp anchors were then re-aligned against the GRCh37 human reference genome using Bowtie 0.12.8 [31], and the resulting alignments were searched for evidence of discordantly aligned anchor pairs. Breakfast then constructs clusters of evidence for chromosomal rearrangements using both discordant read pairs and anchor pairs. To achieve this, the discordant read pairs were reoriented to be in forward-forward orientation. To produce the final list of rearrangement candidates, we filtered out any rearrangements that were not supported by at least 1 paired read and 3 anchor pairs, or by at least 10 anchor pairs.

### RT-PCR and sanger sequencing

Fusion genes were validated using RT-PCR amplification of fusion gene breakpoints of chimeric cDNA and Sanger sequencing. The PCR reactions were 10 min at 95°C; 30 cycles of 30 sec at 95°C, 30 sec at 58–62°C and 30 sec at 72°C and finally 10 min at 72°C. Complementary to the RNA quality control in the RNA-seq and to control the RT-PCR system, primers of β-actin gene were also designed to amplify β-actin simultaneously with fusion gene RT-PCR.

### Fluorescence in situ hybridization of fusion genes

We obtained fluorescence labeled bacterial artificial chromosome (BAC) probes for *LRP1*, *SNRNP25, KCNMB4* and *CCND3*(Empire Genomics) for FISH analysis on frozen sections from the two fusion positive cases [32]. Orange-5TAMRA-dUTP labeled BAC clone RP11-110 J7 was used to identify the *LRP1* gene and green-5TAMRA-dUTP labeled BAC clone CTD-3077 J14 was used to identify the *SNRNP25* gene. For fusion *KCNMB4-CCND3* gene, orange-5TAMRA-dUTP labeled BAC clone RP11-626E3 was used to identify the *KCNMB4* gene and green-5TAMRA-dUTP labeled BAC clone RP11-720D9 was used to identify the *CCND3* gene. Because the genes are located on different chromosomes, overlap of green and orange signals in tumor nucleus indicates gene fusion.

### Western blotting and immunohistochemistry

The detection of possible fusion proteins in osteosarcoma tissue lysate and FFPE tissue sections by western blotting and immunohistochemistry used anti-CCND3, anti-KCNMB4, anti-LRP-1 antibodies (ab28283, ab89703 and ab92544, ABCAM, Cambridge, MA) and anti-SNRNP25 antibody (H00079622-B01, Novus, Littleton, CO).

### Fusion cDNA cloning, cell transformation, stable human osteosarcoma cell transfection, tumor cell proliferation, invasion, migration, and mobility

The cDNA from fusion genes positive cases was PCR amplified with Phusion DNA polymerase (Finnzymes, Fisher Scientific) and EcoRV_LRP1_fw and XhoI_SNRNP25_rev primers. The EcoRV-Xho1 digested fragment was then ligated into a pcDNA3.1(+) vector (Invitrogen) between EcoRV and XhoI restriction sites. The complete cDNA sequences were verified. *KCNMB4-CCND3* was cloned in similar fashion.

Rat2 cells were seeded into a 6-well plate one day before transfection. Solution A contained 50 μl OPTI-MEM medium and 2 μg plasmid DNA with fusion genes or GFP or Ras V12G control vector. Solution B contained of 50 μl OPTI-MEM medium and 5 μl lipofectamine 2000 (Invitrogin). Solutions A and B were mixed and the mixture was kept for 20 min at room temperature, then added to a well containing Rat2 cells in 400 μl OPTI-MEM medium. Transfected Rat2 cells were cultured in regular cell culture conditions with the medium replaced twice a week. The foci were fixed with 3.7% formaldehyde solution for 10 minutes and stained with crystal violet solution for 1 hour and washed with water overnight.

The human osteosarcoma cell line SAOS-2 was obtained from ATCC and maintained in Eagle’s minimum essential medium supplemented with 10% fetal bovine serum and 1% penicillin-streptomycin solution. Cells were incubated at 37°C in a humidified atmosphere of 5% CO_2_. *LRP1*-*SNRNP25* and *KCNMB4-CCND3* constructs were transfected into SAOS-2 cells by lipofectamine 2000 and selected stable cells with 600 μg/ml G418 for 2 weeks. Fusion gene expression was validated by western blotting with anti-CCND3and anti-SNRNP25 antibodies.

Six-well plate with 0.5% agar in medium as the bottom layer was used for soft agar colony formation assay. For each well, 2.5 × 103 cells suspended in medium with 0.375% agarose were plated as top layer and incubated at 37°C for 3 weeks. Three sets were used for each sample. Colonies were stained with 0.005% Crystal violet and counted.

Tumor cell proliferation assay by BrdU incorporation and cell invasion and migration assays by transwell assay were performed as previously described [29].

## Additional file

Additional file 1:
**The clinical information of 11 human osteosarcoma patients and the results of osteosarcoma transcriptome sequencing data, including the fusion gene list, fusion gene structures, fusion gene validations by PR-PCR, p53 mutations, fusion gene transfection and functional studies.**
