# Supplementary material for: Recurrent LRP1-SNRNP25 and KCNMB4-CCND3 fusion genes promote tumor cell motility in human osteosarcoma
Source: J Hematol Oncol. 2014 Oct 10;7:76. doi: 10.1186/s13045-014-0076-2 (PMC4197299; doi:10.1186/s13045-014-0076-2)
Supplement: Additional file 1: — The clinical information of 11 human osteosarcoma patients and the results of osteosarcoma transcriptome sequencing data, including the fusion gene list, fusion gene structures, fusion gene validations by PR-PCR, p53 mutations, fusion gene transfection and functional studies. [file 13045_2014_76_MOESM1_ESM.docx]

Table S1. Clinical information of 11 osteosarcoma patients.

| **MDID** | **Path ID** | **Sample** | **Gender** | **Age** | **Tumor site** | **Pathological diagnosis** |
| --- | --- | --- | --- | --- | --- | --- |
| **226920** | 318924 | **10** | Male | 46 | C40.2, femur metaphysis | Conventional osteosarcoma |
| **217089** | 308436 | **3-1** | Female | 28 | C76.3, iliac | Conventional osteosarcoma |
| **215559** | 309086 | **8** | Female | 16 | C40.2, femur metaphysis | Parosteal osteosarcoma |
| **212382** | 304008 | **11-2** | Male | 18 | C40.2, femur metaphysis | Conventional osteosarcoma |
| **209101** | 302176 | **4** | Male | 48 | C40.0, humerus metaphysis | Conventional osteosarcoma |
| **207220** | 305492 | **2** | Female | 20 | C40.2, tibia metaphysis | Conventional osteosarcoma |
| **206278** | 311857 | **1** | Male | 22 | C40.2,femur metaphysis | Conventional osteosarcoma |
| **199998** | 295666 | **6-2** | Male | 19 | C40.2, tibia metaphysis | Conventional osteosarcoma |
| **168548** | 311138 | **9** | Female | 21 | C40.2, femur metaphysis | Conventional osteosarcoma |
| **159384** | 260401 | **3-3** | Male | 46 | C76.3, iliac | Conventional osteosarcoma |
| **146318** | 248231 | **6-1** | Female | 16 | C40.2, femur metaphysis | Conventional osteosarcoma |

**Table S2. Fusion genes detected in our cohort of 11 human osteosarcomas.**

| **Fusion gene** | **5' breakpoint** | **3' breakpoint** | **Predicted consequence** | **Predicted mechanism** | **Positive samples** |
| --- | --- | --- | --- | --- | --- |
| *LRP1-SNRNP25* | chr12:57548484 (+) | chr16:105459 (+) | Chimeric protein | Interchromosomal translocation | 1 |
| *TP53-AC016582.2* | chr17:7590695 (-) | chr19:38327655 (-) | Loss-of-function | Interchromosomal translocation | 1 |
| *DUSP14-BCAS3* | chr17:35850064 (+) | chr17:59024580 (+) | Loss-of-function | Deletion | 1 |
| *FAM120C-PAGE1* | chrX:54208933 (-) | chrX:49458804 (-) | Chimeric protein | Deletion | 1 |
| *AUTS2-GLI3* | chr7:70136606 (+) | chr7:42066011 (-) | Loss-of-function | Inversion | 3-3 |
| *GAPVD1-COL5A1* | chr9:128024238 (+) | chr9:137619112 (+) | Loss-of-function | Deletion | 3-3 |
| *KMT2C-AC006372.4* | chr7:152007051 (-) | chr7:157263563 (+) | Loss-of-function | Inversion | 4 |
| *TP53-CCNB1* | chr17:7590695 (-) | chr5:68463735 (+) | Loss-of-function | Interchromosomal translocation | 6-2 |
| *PRR5-SYT13* | chr22:45128271 (+) | chr11:45277442 (+) | Chimeric protein | Interchromosomal translocation | 6-2 |
| *HMGA2-MSRB3* | chr12:66232349 (+) | chr12:65856935 (+) | Chimeric protein | Tandem duplication | 8 |
| *ZFC3H1-MDM2* | chr12:72050665 (-) | chr12:69218143 (+) | Chimeric protein | Inversion | 8 |
| *ERG-IFNGR2* | chr21:40033582 (-) | chr21:34787195 (+) | Loss-of-function | Interchromosomal translocation | 8 |
| *KCNMB4-CCND3* | chr12:70760850 (+) | chr6:41908323 (-) | Loss-of-function | Interchromosomal translocation | 9 |
| *MDM2-RUNX2* | chr12:69222711 (+) | chr6:45296398 (+) | Loss-of-function | Interchromosomal translocation | 9 |
| *IRAK3-RUNX2* | chr12:66583212 (+) | chr6:45399600 (+) | Loss-of-function | Interchromosomal translocation | 9 |
| *DPM1-CD63* | chr20:49571723 (-) | chr12:56121123 (-) | Chimeric protein | Interchromosomal translocation | 10 |

Table S3. the fusion gene specific primers used in the RT-PCR validation.

| ID | Gene | Primer sequences (5'to3') | locations | Size |
| --- | --- | --- | --- | --- |
| Primer 1 | *TP53* | *TGCTCAAGACTGGCGCTAAA* | Exon 1 | 190bp |
| Primer 2 | *CCNB1* | *GCCATGTTGATCTTCGCCTT* | Exon 2 |  |
| Primer 3 | *MDM2* | *CTGGCTCTGTGTGTAATAAGG* | Exon 9 | 156bp |
| Primer 4 | *RUNX2* | *CTGTTTGATGCCATAGTCCC* | Exon 2 |  |
| Primer 5 | *DPM1* | *ggaagcccagatggaacaag* | Exon 2 | 196bp |
| Primer 6 | *CD63* | *ggaagaggaagacacccact* | Exon 3 |  |
| Primer 7 | *LRP1* | *CTGGTCAGCCGCCTTGTCTAC* | Exon 8 | 145bp |
| Primer 8 | *SNRNP25* | *Cgtattctagggctatttggg* | Exon 2 |  |
| Primer 9 | *KCNMB4* | *GCACGCCAGCCGCCGAGAGT* | Exon 1 | 447bp |
| Primer 10 | *CCND3* | *TGCAGAATGAAGGCCAGGAA* | Exon 4 |  |
| Primer 11 | *ZFC3H1* | *GAGGACCAAACTAGCACTGA* | Exon 2 | 285bp |
| Primer 12 | *MDM2* | *CTTTTGATCACTCCCACCTT* | Exon 7 |  |

Table S4. Scanning ***LRP-1-SNRNP25*** and ***KCNMB4-CCND3*** in 271 sarcomas including osteosarcoma

| Sarcoma types | Cases | Fusion gene positive frequency |
| --- | --- | --- |
| osteosarcoma | 31 | 2 (2/31, 6.5%, *LRP-1-SNRNP25*) |
|  |  | 2 (2/31,6.5%, *KCNMB4-CCND3*) |
| MFH/UPS | 56 | 0 |
| Liposarcoma | 50 | 0 |
| Leiomyosarcoma | 24 | 0 |
| Rhabdomyosarcoma | 4 | 0 |
| Synovial sarcoma | 21 | 0 |
| Chondrosarcoma | 13 | 0 |
| Ewing sarcoma | 8 | 0 |
| MPNST | 64 | 0 |
| Total: | 271 |  |


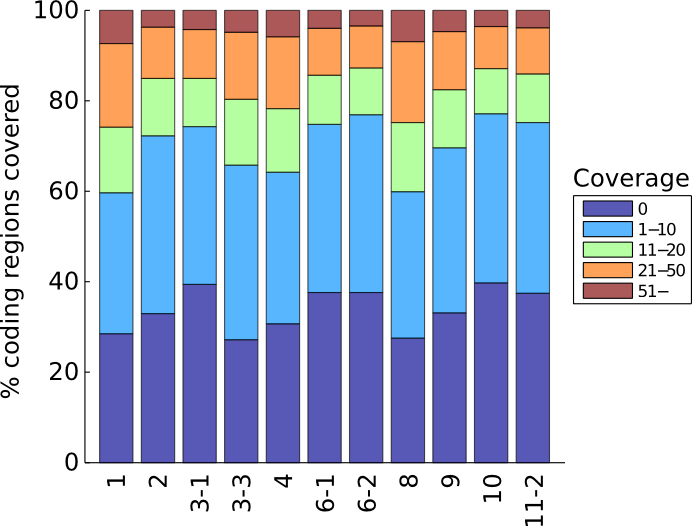


Figure S1. Percentage of bases within coding regions covered by at least 0, 1, 11, 21 or 51 reads.

Data is shown for all 11 osteosarcoma patients in our sequencing cohort.


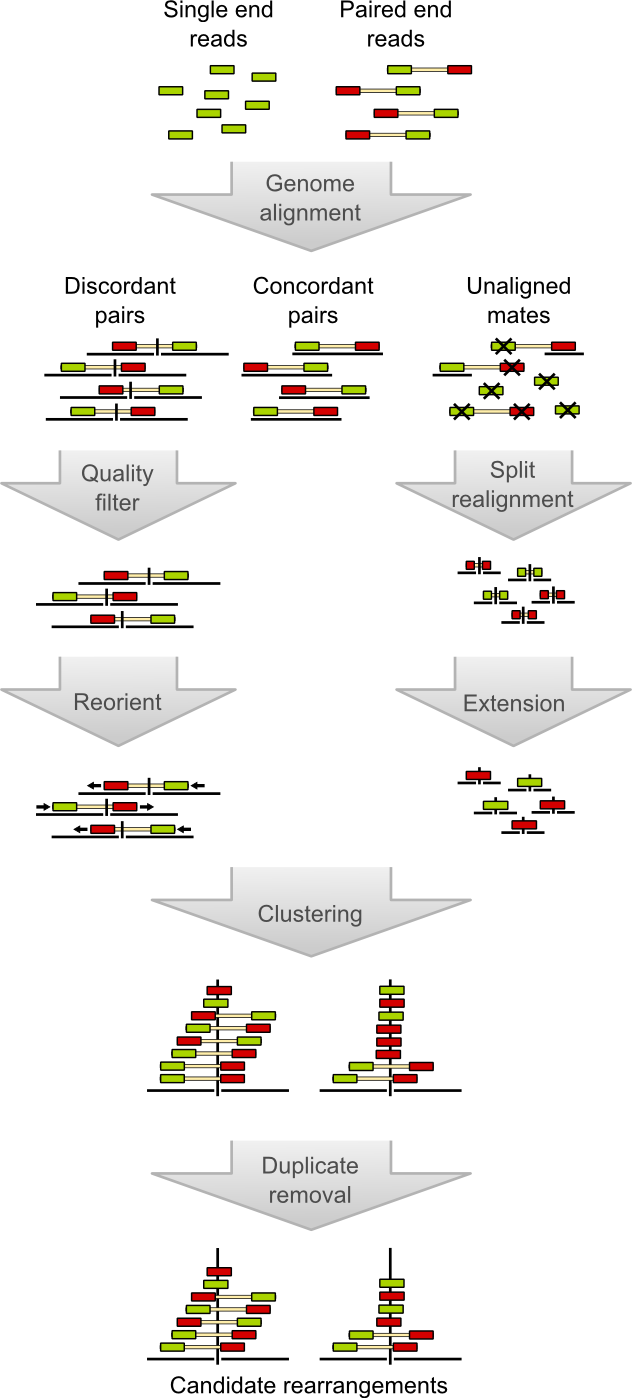


Figure S2. Illustration of the fusion discovery method employed by our fusion gene discovery tool, Breakfast.

All fusion genes described in this paper were discovered by both Breakfast and Chimerascan, a third party fusion gene detection software.


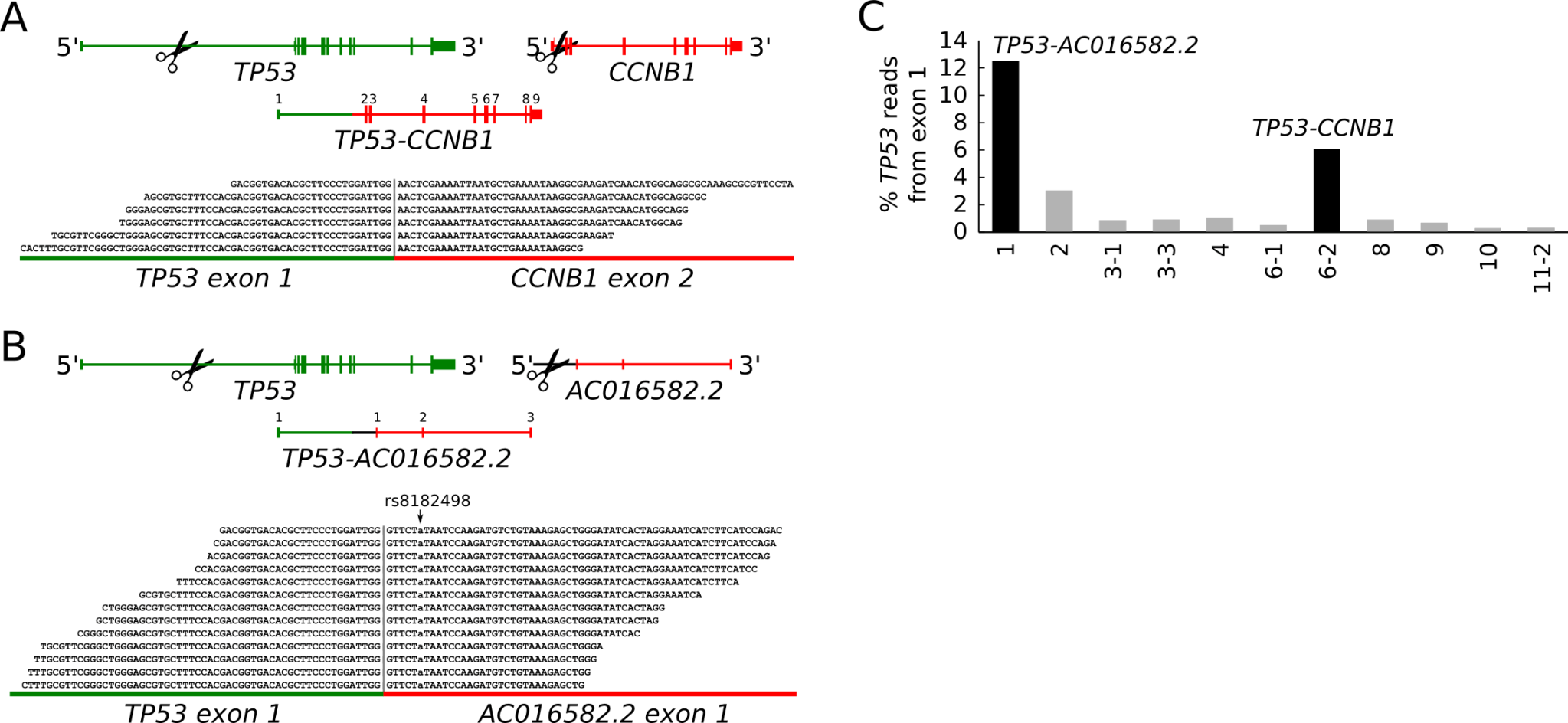


Figure S3. ***TP53***-disrupting rearrangements were found in two osteosarcomas in our cohort.

(A-B) Structure of the *TP53-CCNB1* and *TP53-AC016582.2* fusion genes found in samples 6-2 and 1, respectively. Reads overlapping the fusion junction are shown in the bottom panels. (C) The two *TP53-*rearranged tumors displayed a higher fraction of reads aligned to exon 1, relative to reads aligned to all *TP53* exons. Exon expression beyond the first exon is disrupted by the rearrangements.


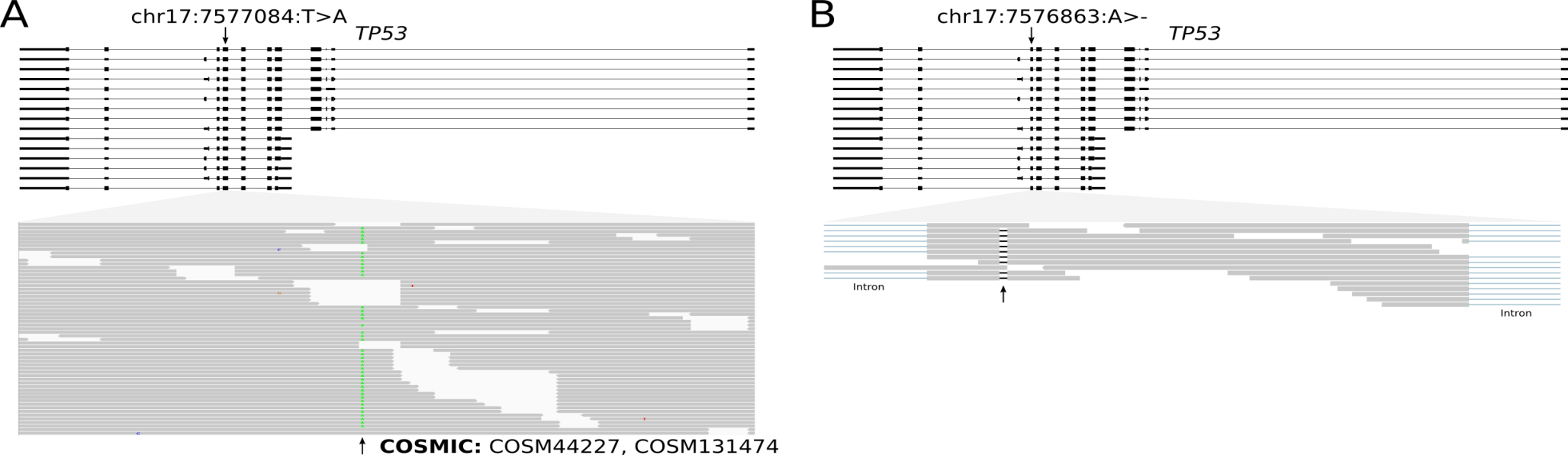
Figure S4. *T****P53*** mutations in human osteosarcoma samples 3-1 and 3-3.

(A-B) Top panel shows the structures of *TP53* transcript variants and the location of the mutation. Bottom panel shows the read level evidence for the mutant allele, indicating loss-of-heterozygosity in both cases.

Figure S5. Evidence for ***TP53*** deletions in human osteosarcoma samples 2 and 6-1. (A) Transcriptome reads extracted from sample 2 span a 250 kb deleted region containing *TP53*. T
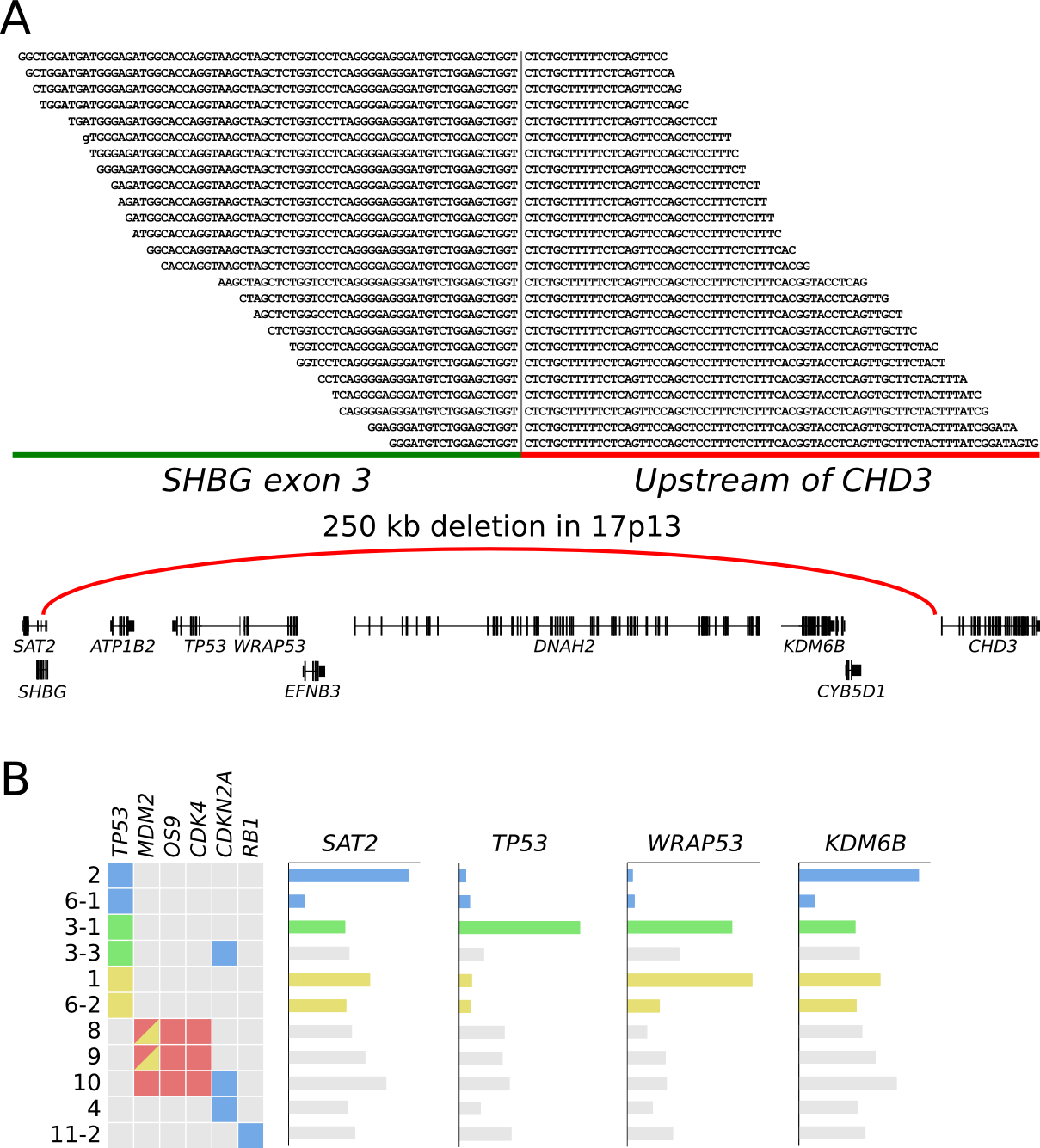
op panel displays individual reads that bridge the junction (red connecting line) created by the deletion. (B) In sample 6-1, a localized gene dosage effect in 17p13 suggests a deletion.


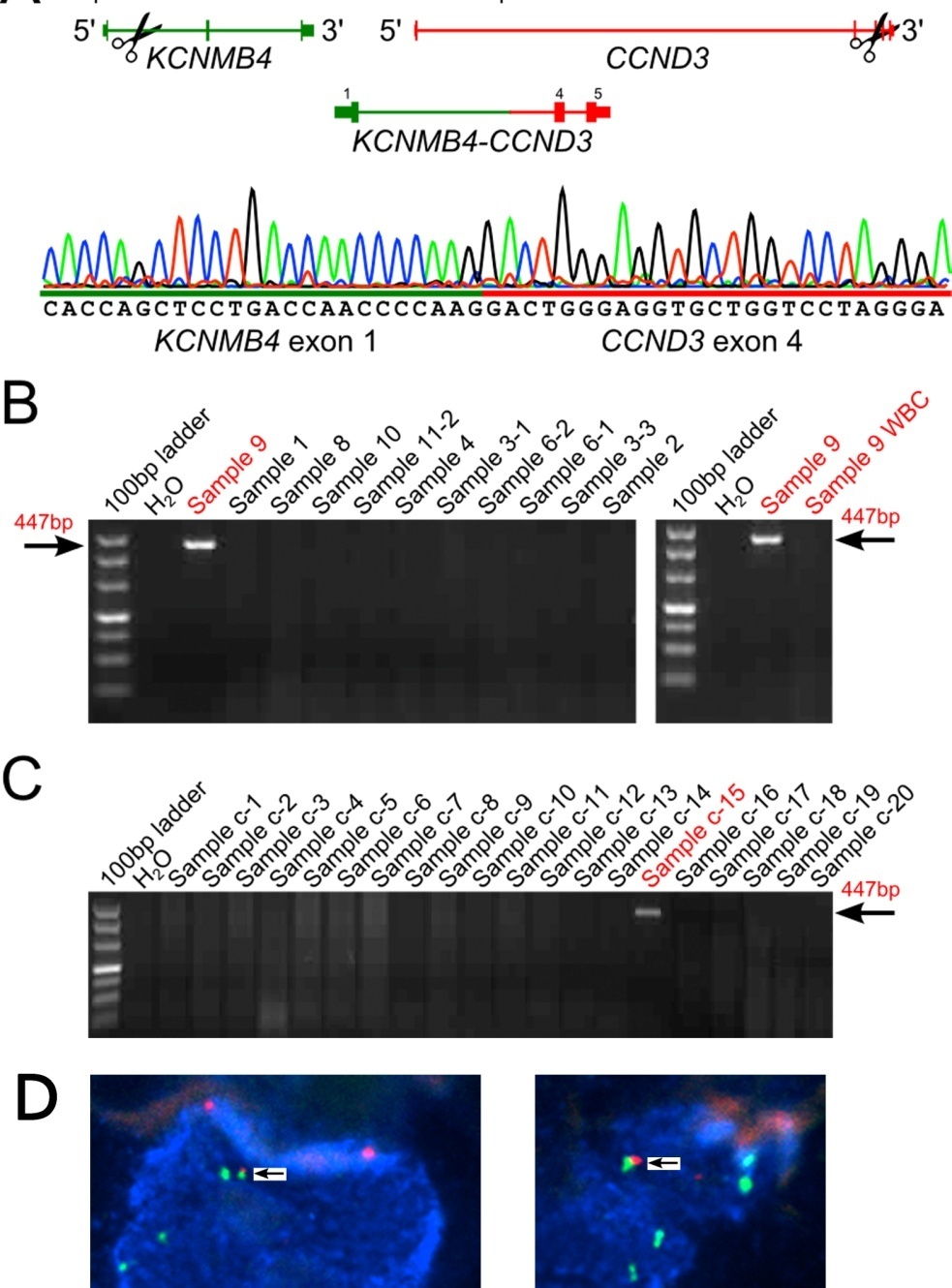


**Figure S6. Structure and validation of the *KCNMB4-CCND3* fusion gene*.***(A) Structure of the fusion gene based on transcriptome sequencing. Fusion transcript was validated with Sanger sequencing. Transcript variants shown are *NM_014505* for *KCNMB4* and *NM_001760* for *CCND3*. These are the transcript variants with the highest expression in osteosarcoma cells. (B) RT-PCR validation of fusion transcript in the sequencing cohort. No evidence for fusion was found in normal white blood cells (WBC) of the fusion positive patient. (C) RT-PCR identified a second fusion positive case in a validation cohort of 20 osteosarcomas. (D) FISH method detection validated the fusion gene of *KCNMB4* *CCND3*. Arrows indicated overlapping probes.


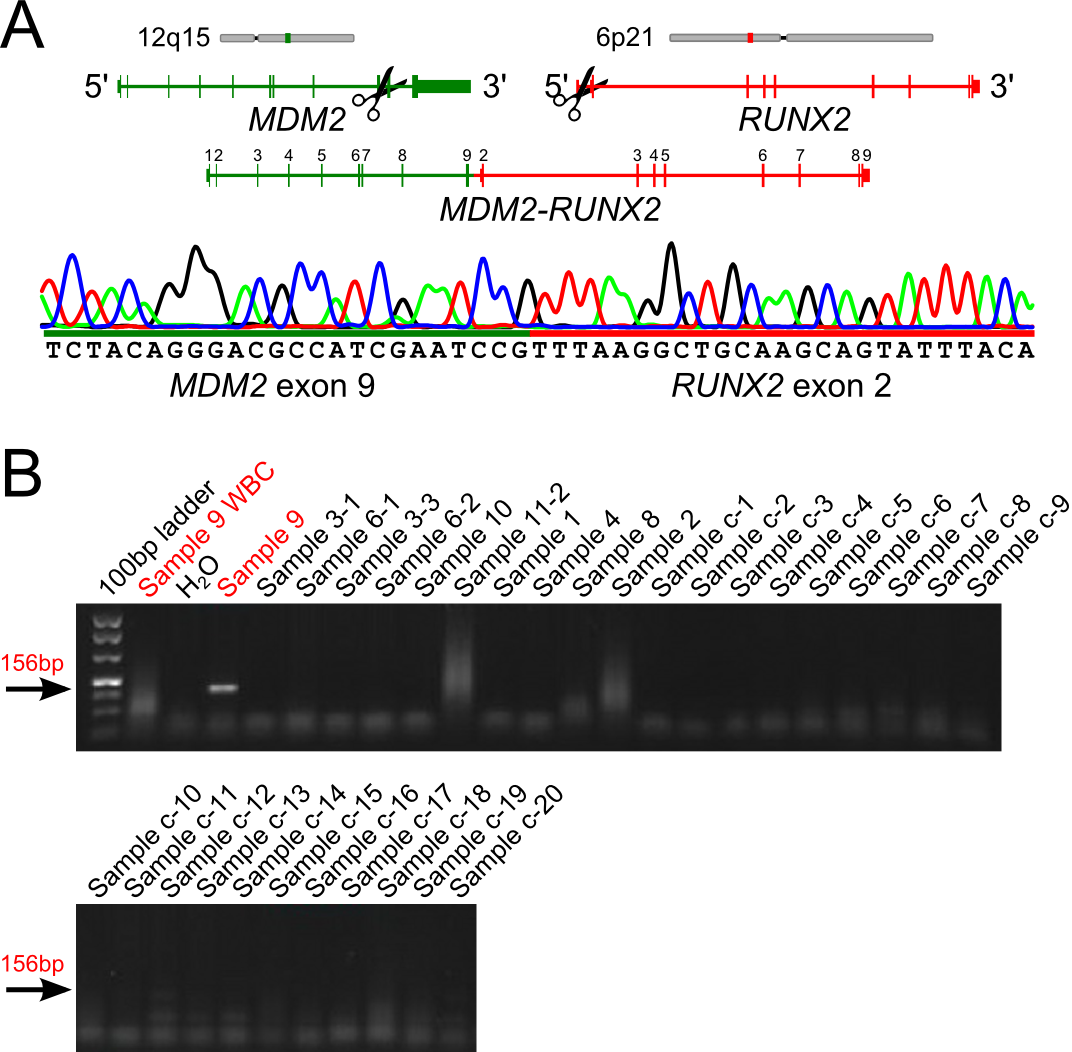


Figure S7. Structure and validation of the ***MDM2-RUNX2*** fusion gene***.***(A) Structure of the fusion gene based on transcriptome sequencing. Fusion transcript was validated with Sanger sequencing. Transcript variants shown are *NM_002392* for *MDM2* and *NM_001015051* for *RUNX2*. These are the transcript variants with the highest expression in osteosarcoma cells. (B) RT-PCR validation of fusion transcript in the sequencing cohort and in a validation cohort of 20 osteosarcomas. No evidence for fusion was found in normal white blood cells (WBC) of the fusion positive patient.


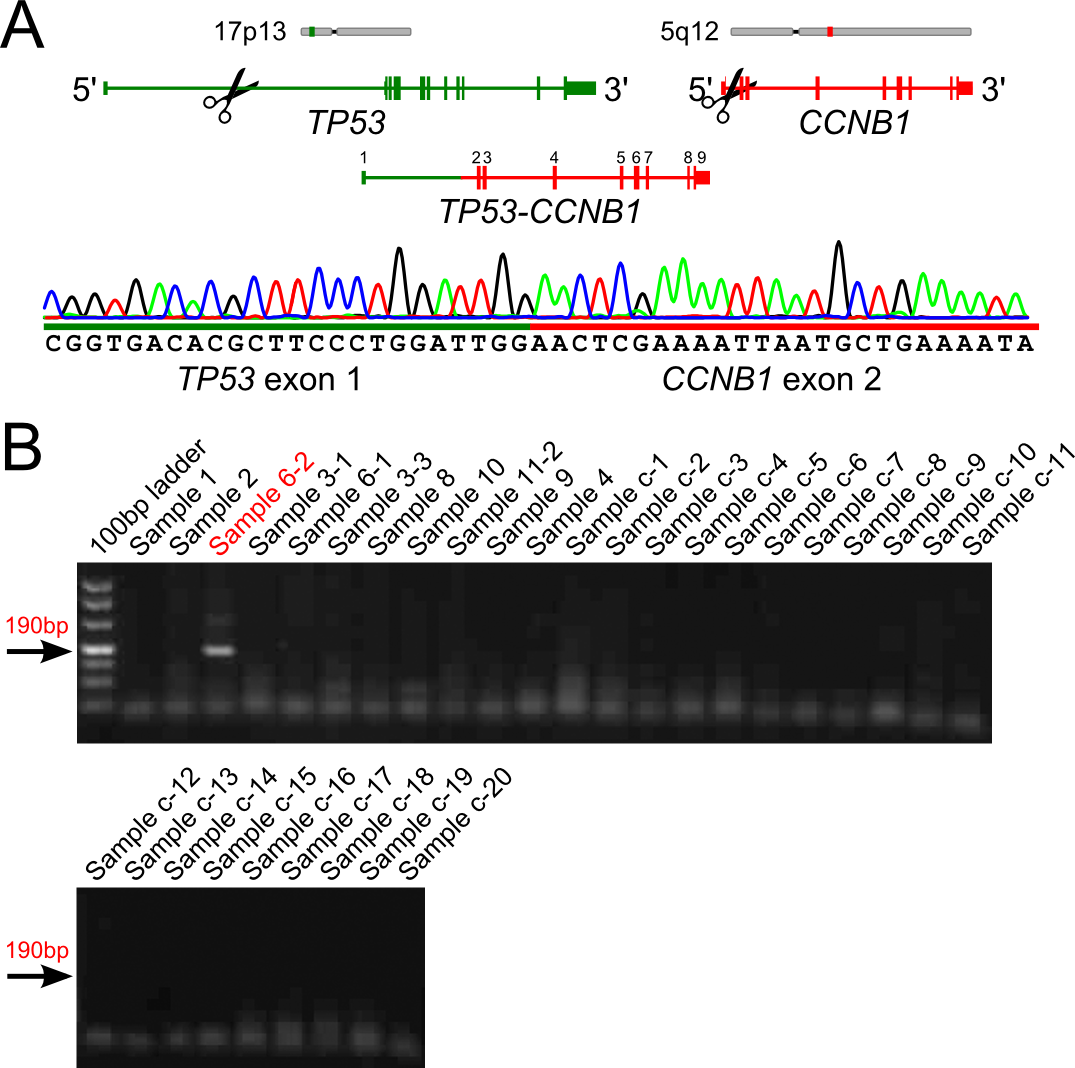


Figure S8. Structure and validation of the ***TP53-CCNB1*** fusion gene***.***(A) Structure of the fusion gene based on transcriptome sequencing. Fusion transcript was validated with Sanger sequencing. Transcript variants shown are *NM_001276761* for *TP53* and *NM_031966* for *CCNB1*. These are the transcript variants with the highest expression in osteosarcoma cells. (B) RT-PCR validation of fusion transcript in the sequencing cohort and in a validation cohort of 20 osteosarcomas.


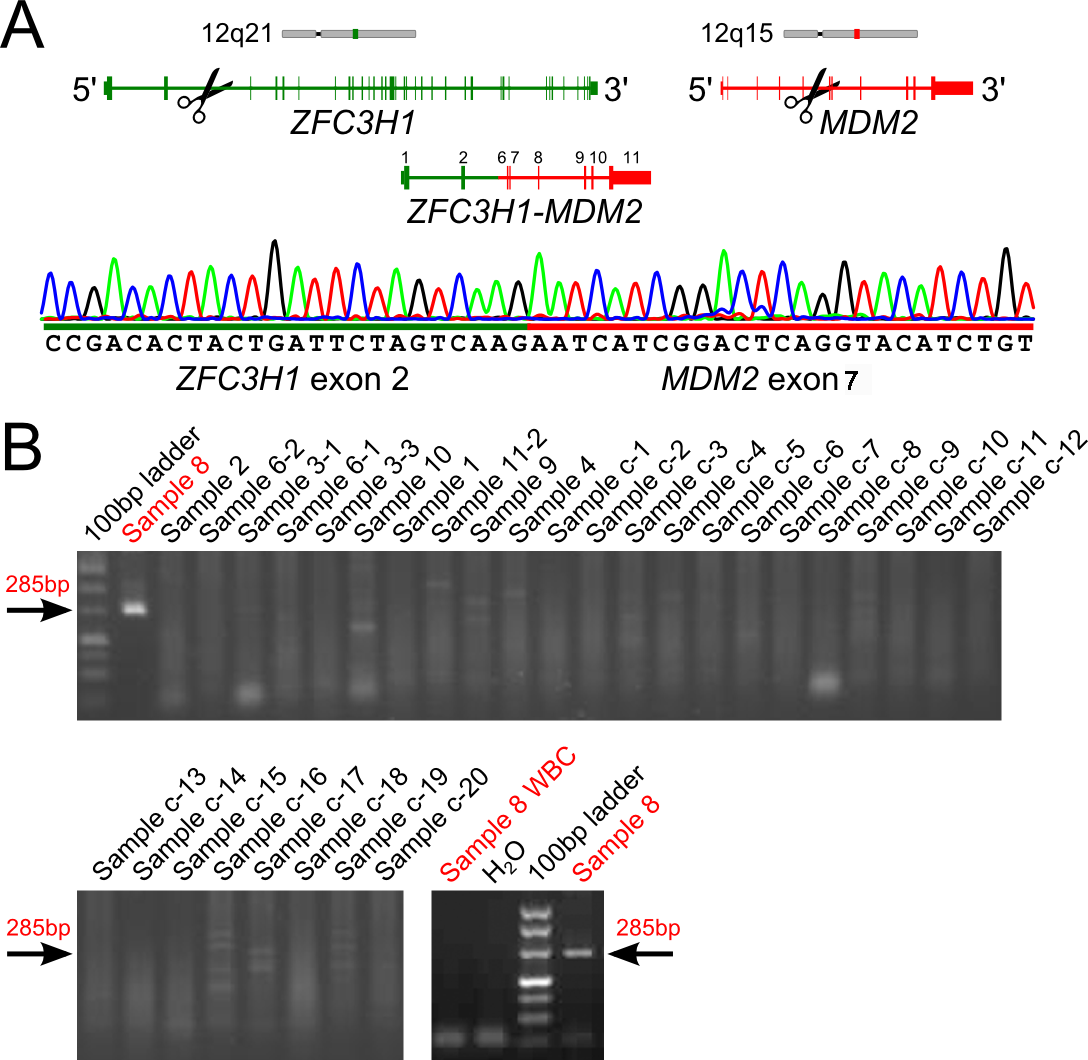


Figure S9. Structure and validation of the ***ZFC3H1-MDM2*** fusion gene***.***(A) Structure of the fusion gene based on transcriptome sequencing. Fusion transcript was validated with Sanger sequencing. Transcript variants shown are *NM_002392* for *MDM2* and *NM_144982* for *ZFC3H1*. These are the transcript variants with the highest expression in osteosarcoma cells. (B) RT-PCR validation of fusion transcript in the sequencing cohort and in a validation cohort of 20 osteosarcomas. No evidence for fusion was found in normal white blood cells (WBC) of the fusion positive patient.


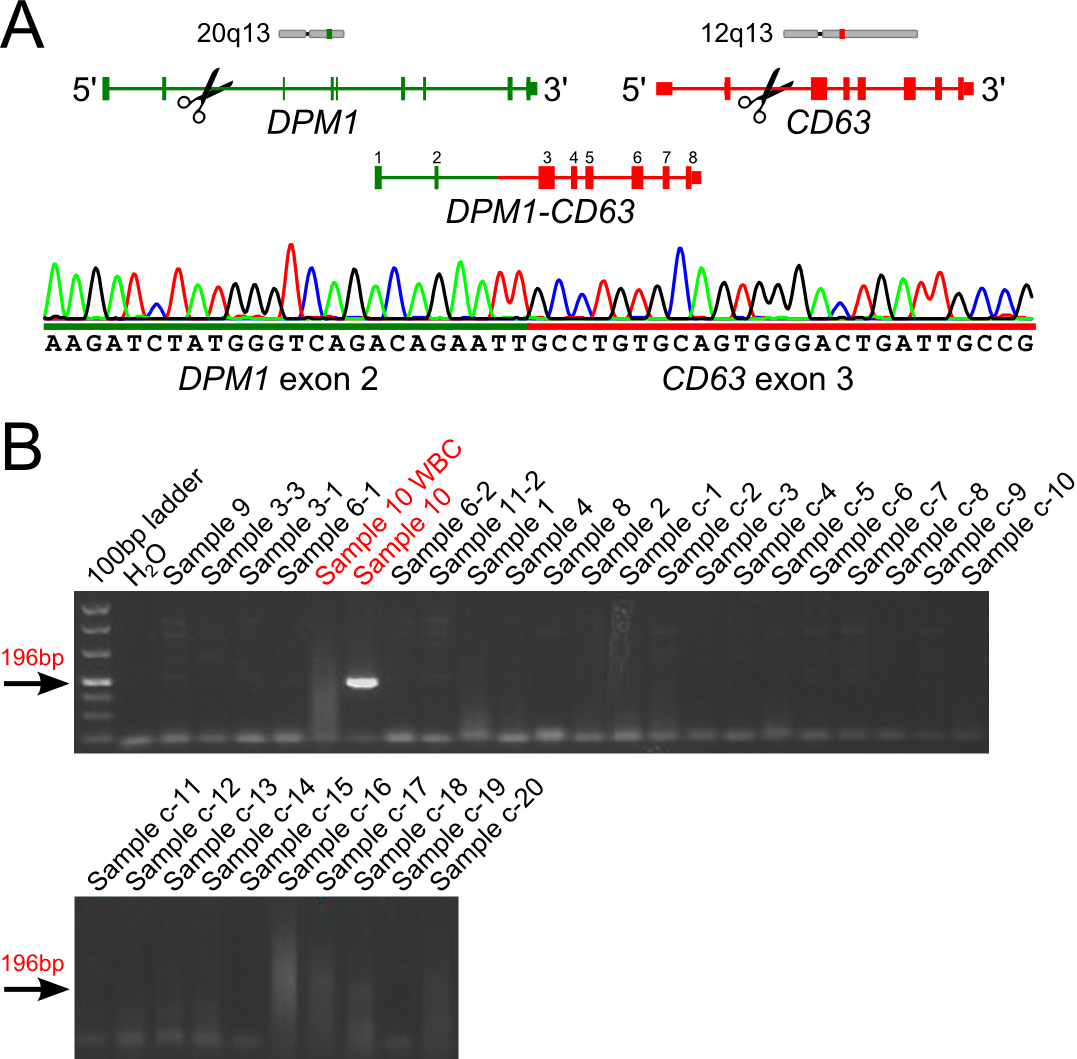


Figure S10. Structure and validation of the ***DPM1-CD63*** fusion gene***.***(A) Structure of the fusion gene based on transcriptome sequencing. Fusion transcript was validated with Sanger sequencing. Transcript variants shown are *NM_003859* for *DPM1* and *NM_001780* for *CD63*. These are the transcript variants with the highest expression in osteosarcoma cells. (B) RT-PCR validation of fusion transcript in the sequencing cohort and in a validation cohort of 20 osteosarcomas. No evidence for fusion was found in normal white blood cells (WBC) of the fusion positive patient.


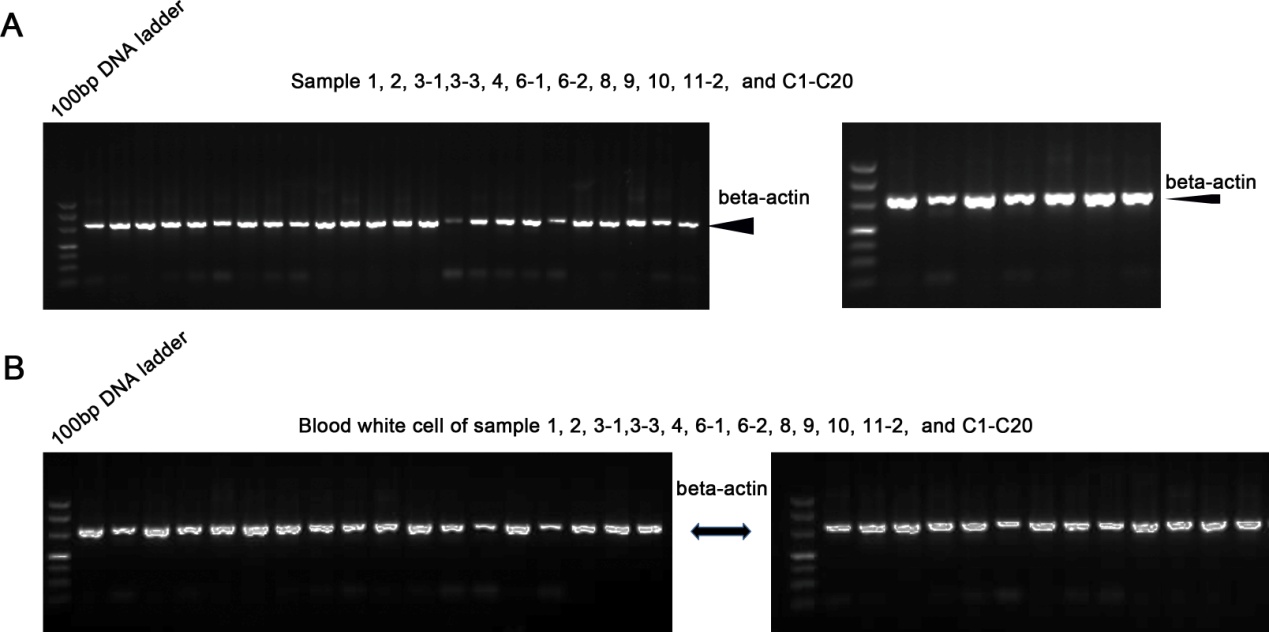


Figure S11. RT-PCR of ***β-actin*** from the RNA of 31 osteosarcoma samples and blood white cells. (A): fresh tissue samples. (B): blood white cell samples.


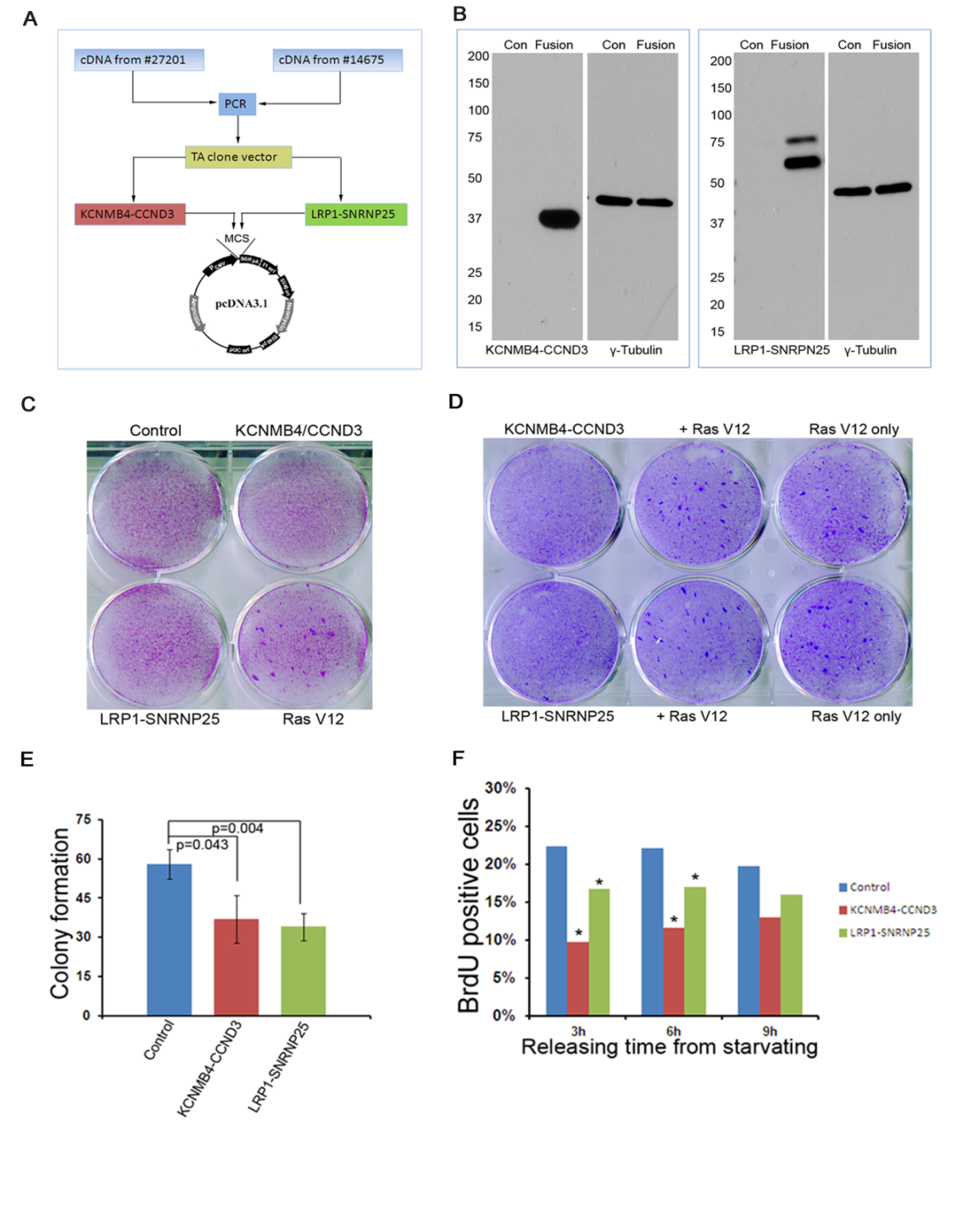


Figure S12. ***LRP1-SNRNP25*** and ***KCNMB4-CCND3*** fusion genes are not sufficient for initiating cell transformation and promoting cell proliferation. The clones of ***LRP1-SNRNP25*** and ***KCNMB4-CCND3*** fusion genes into pcDNA3.1 express vector and overexpression of the fusion genes in the stable transfected human osteosarcoma SAOS-2 cells.

(A) Cloning strategy used for ***LRP1-SNRNP25*** and ***KCNMB4-CCND3*** fusion genes. (B) Validation of the fusion genes expression in the stable transfected clones. The expression of the fusion genes were detected by western blotting with anti-CCND3 antibody and anti-SNRPN25. The fusion gene products were shown with the expected molecular weight. (C) ***LRP1-SNRNP25*** and ***KCNMB4-CCND3*** fusion genes failed to induce Rat2 cell transformation. (D) ***LRP1-SNRNP25*** and ***KCNMB4-CCND3*** fusion genes did not enhance ***K-Ras V12G*** induced Rat2 cell transformation. (E) ***LRP1-SNRNP25*** and ***KCNMB4-CCND3*** fusion genes inhibited colony formation in soft agar. (F) ***LRP1-SNRNP25*** and ***KCNMB4-CCND3*** fusion genes inhibited cell proliferation. The stable SAOS-2 cells with the fusion genes were starved in FBS-free medium for 4 days, the cells were released from the starvation by using fresh medium for 0h, 3h, 6h and 9 hours and add 10 mM BrdU for one hour before harvesting the cells, the percentage of BrdU positive cells were quantified by flow cytometry, a representative data was shown in three independent experiments. * p < 0.05.
